# MicroRNA-Based Liquid Biopsy for Cervical Cancer Diagnostics and Treatment Monitoring

**DOI:** 10.3390/ijms252413271

**Published:** 2024-12-10

**Authors:** Maria A. Kepsha, Angelika V. Timofeeva, Vasiliy S. Chernyshev, Denis N. Silachev, Elena A. Mezhevitinova, Gennadiy T. Sukhikh

**Affiliations:** National Medical Research Center for Obstetrics, Gynecology and Perinatology Named After Academician V.I. Kulakov, Ministry of Healthcare of the Russian Federation, Moscow 117997, Russiasilachevdn@belozersky.msu.ru (D.N.S.);

**Keywords:** cervical cancer, diagnosis, biomarkers, treatment monitoring, microRNA, miRNA, extracellular vesicles, exosomes

## Abstract

Despite prevention strategies, cervical cancer remains a significant public health issue. Human papillomavirus plays a critical role in its development, and early detection is vital to improve patient outcomes. The incidence of cervical cancer is projected to rise, necessitating better diagnostic tools. Traditional screening methods like the cytological examination and human papillomavirus testing have limitations in sensitivity and reproducibility. Liquid-based cytology offers some improvements, but the need for more reliable and sensitive techniques persists, particularly for detecting precancerous lesions. Liquid biopsy is a non-invasive method that analyzes cancer-derived products in biofluids like blood, offering potential for real-time monitoring of tumor progression, metastasis, and treatment response. It can be based on detection of circulating tumor cells (CTCs), circulating free DNA (cfDNA), and microRNAs (miRNAs). This review particularly underlines the potential of microRNAs, which are transported by extracellular vesicles. Overall, this article underscores the importance of continued research into non-invasive diagnostic methods like liquid biopsy to enhance cervical cancer screening and treatment monitoring.

## 1. Introduction

Cervical cancer (CC) remains a major public health concern for middle-aged women with a clear socioeconomic gradient; incidence and mortality are significantly higher in low-HDI (Human Development Index) countries compared to very high-HDI countries. While global incidence has generally declined, stabilizing at low levels in high-income countries since 2005, it has risen in parts of eastern Africa and eastern Europe [1,2]. More than 80% of women and over 90% of men are known to be infected with the human papillomavirus (HPV) during their lifetime, typically by the age of 45 [3]. This infection resolves spontaneously in most women within a few years; however, HPV can persist and cause slow progressing changes in the cervix, which eventually lead to CC [4]. According to the World Health Organization (WHO), the annual incidence of CC is projected to increase from 570,000 to 700,000 between 2018 and 2030 and the annual mortality is expected to rise from 311,000 to 400,000 cases. In response, the WHO launched a global strategy to accelerate the elimination of CC as a public health problem, which aims to reduce the incidence of CC cases to 4 per 100,000 women-years [2,5].

Clinical practice of early diagnosis plays a pivotal role in combating malignant diseases, facilitating timely treatment and increasing the likelihood of patient recovery [6]. In Russia, the prevention of CC is implemented through programs of organized and opportunistic CC screening, which include cytological examination (Pap smear or liquid-based cytology), HPV testing, or a combination of both (co-testing). For decades, the Pap test has been the primary screening method, demonstrating high efficacy in reducing the incidence of and mortality from CC [7,8,9,10]. Published studies report a high specificity (86–100%) of the oncocytopathological method, yet its limited reproducibility and sensitivity (30–87%) present a significant risk of missing precancerous lesions [11,12]. According to Gupta et al., discrepancies between cytological and subsequent histological diagnoses occur in 27% of cases [13]. A more advanced cytological method is liquid-based cytology (LBC). LBC allows identifying cellular composition of the exo- and endocervix, thereby reducing the proportion of unsatisfactory smears and also facilitate HPV testing and the assessment of p16/Ki67 oncoprotein expression. Nevertheless, studies comparing the sensitivity of conventional Pap smear and LBC yield conflicting results regarding whether LC is more [14,15] or equally [16,17,18] sensitive compared to the Pap test. The identification of the etiological link between HPV and the development of CC has led to the implementation of DNA testing as a primary screening method [19,20,21]. A multinational cohort study (*n* = 24,295) demonstrated that the primary HPV screening has high sensitivity (90%) for detecting high-grade squamous intraepithelial lesions (HSIL) and CC [22]. On the other hand, in younger women, the HPV DNA test has lower specificity and a 22% lower positive predictive value than cytology. This is due to the transient nature of papillomavirus infection, leading to unwarranted diagnostic and therapeutic interventions [23].

**Figure 1:**
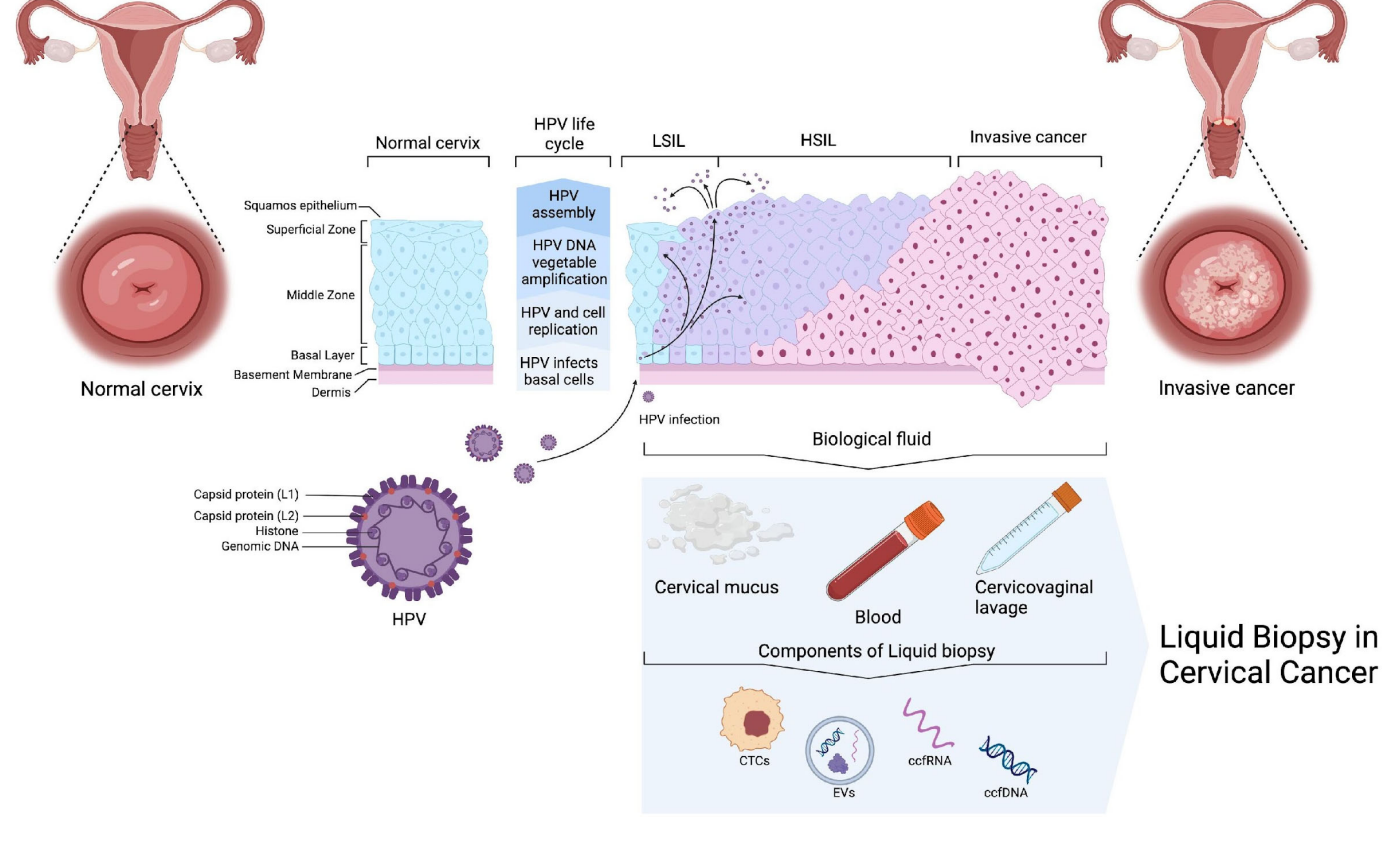
Illustration summarizing the stages of CC progression with comparison to a normal cervix and highlighting biofluids that can potentially be used for liquid biopsy. HPV—human papillomavirus; LSIL—low-grade squamous intraepithelial lesions; HSIL—high-grade squamous intraepithelial lesions; CTCs—circulating tumor cells; EVs—extracellular vesicles; ccfRNA—cell-free circulating RNA; ccfDNA—cell-free circulating DNA.

Tissue biopsy is the gold standard for diagnosing neoplasms, including squamous lesions of the cervix. This method allows for the determination of the tumor’s morphological type and its molecular–genetic characteristics, which aids in defining management strategies and treatment of patients [24]. However, the traditional tissue biopsy is an invasive procedure associated with risks for patients and technical difficulties in sample collection [25]. Liquid biopsy may be useful for diagnosing recurrence in patients who have already undergone treatment for CC, as traditional biopsy procedures may be challenging, particularly in metastatic sites. Therefore, the development and implementation of non-invasive strategies for accurate and early cancer diagnosis and monitoring are of great importance. A promising diagnostic method is liquid biopsy (LB), which is based on the detection of levels of circulating tumor cells (CTCs), free circulating tumor DNA (ctDNA), coding and non-coding RNAs, and extracellular vesicles (EVs) in blood and other biological fluids (Figure 1) [26]. In recent years, interest in LB has increased due to its significant potential in the early diagnosis of cancer and in predicting its progression [27]. LB is a non-invasive or minimally invasive method that allows for real-time acquisition of information on tumor heterogeneity, early detection of metastases, and identification of minimal residual disease [28]. Additionally, the non-invasive nature of liquid biopsies allows for repeated testing, disease monitoring, and the assessment of treatment responses and resistances with minimal patient discomfort.

## 2. Liquid Biopsy in Cervical Cancer

LB is emerging as a potential new method for diagnosing CC by analyzing cancer cells or cancer biomarkers released into extracellular space and that are present in biofluids such as blood (plasma and serum), cervical mucus, and cervicovaginal lavage (Figure 1).

### 2.1. Circulating Tumor Cells (CTCs)

Collecting circulating tumor cells (CTCs) offers a non-invasive way to study tumor heterogeneity, monitor disease progression, and assess treatment effectiveness, and they serve as a negative prognostic factor for CC [29]. Studies show that the presence and count of CTCs correlate with shorter progression-free survival (PFS) and can serve as prognostic markers and indicators of treatment efficacy in CC, particularly in advanced stages [30]. For example, a decrease in CTC count after treatment with bevacizumab, an anti-angiogenesis drug, was linked to improved survival outcomes. This suggests that CTCs can serve as a predictive biomarker for treatment response, particularly in therapies targeting angiogenesis [31]. Despite their potential, there are challenges in utilizing CTCs for CC management. This is likely due to the absence of specific tumor markers and the low concentration of CTCs in peripheral blood samples. The variability in detection methods and the need for further validation in larger cohorts are significant hurdles. Additionally, while CTCs provide valuable insights into the metastatic potential and treatment response, their role in early detection and routine clinical application requires further research and technological advancements.

### 2.2. Circulating Cell-Free DNA (ccfDNA)

Circulating cell-free DNA (ccfDNA), particularly circulating cell-free HPV DNA (ccfHPV DNA), is gaining attention as a promising biomarker for CC [32]. This approach involves analyzing DNA fragments released into the bloodstream by tumor cells, offering a non-invasive method for disease detection and monitoring. Increased ccfDNA levels were correlated with FIGO (The International Federation of Gynecology and Obstetrics), tumor stage, histological grade, infiltration depth, and the presence of lymphatic metastases [33].

Zhang and colleagues [34] conducted one of the largest ccfDNA studies, which included over 10,000 Chinese patients across various cancer types, including 126 cases of CC. Using a next-generation sequencing (NGS) panel covering 1020 genes, PIK3CA was identified as the most frequently mutated gene (about 30%), followed by MLL3, TP53, and others. Based on these findings, which closely align with previously reported CC tissue sequencing results [35], the authors advocate for the global use of ccfDNA in precision medicine initiatives. Given the association between CC and HPV, some researchers have concentrated their efforts on investigating cell-free HPV DNA as a potential tumor marker [36]. Sivars and coworkers found ccfHPV DNA in 94.4% of pre-treatment plasma samples from patients with locally advanced CC. Persistent ccfHPV DNA at the end of treatment was correlated with worse progression-free survival than if ccfHPV DNA was cleared [37]. In a more recent study, Han et al. clinically validated that circulating tumor HPV DNA can be used for optimizing follow-up of patients with HPV-associated cervical carcinoma at high risk of recurrence. This highlights the fact that HPV DNA can potentially be used to identify highly personalized tumor markers and perform the most effective therapy [32].

While ccfDNA offers promising applications in the diagnosis and management of CC, there are several challenges associated with its use. For instance, depending on the type and stage of the disease, ccfDNA can be present at low concentrations and have short half-life (5–150 min), necessitating rapid and precise processing to obtain accurate results [38,39]. Furthermore, ccfDNA is frequently fragmented into shorter lengths, making it susceptible to chemical damage. This fragmentation can impede the ability to accurately detect and quantify ccfDNA, affecting overall performance of the tests [38,40]. While it was shown to be very sensitive and specific in early-stage invasive carcinoma, ccfDNA analysis may have limited sensitivity in detecting HPV ccfDNA in pre-invasive carcinoma [41]. The described limitations can reduce its effectiveness as a standalone early screening tool for all carcinoma subtypes, which necessitates its use in conjunction with other diagnostic methods.

### 2.3. Cell-Free Circulating RNA

#### 2.3.1. Coding RNAs

mRNA is a type of coding RNA that carries genetic information from DNA to the ribosome, where proteins are synthesized. In the context of circulating mRNA, these molecules are released into the bloodstream as the result of tumor cell necrosis, apoptosis, and active release by cancer cells [42]. In 1997, Pao et al. identified the presence of CC cells in the peripheral blood of stage IVb CC patients with distant metastasis. HPV type 16 DNA was identified in 13 out of 15 CC tissue samples, and HPV-specific mRNA was found in the blood of 12 of these 13 patients, indicating the presence of circulating cancer cells. These findings suggest that the detection of HPV E6 mRNA in peripheral blood could serve as a sensitive marker for circulating CC cells and may have clinical relevance in monitoring disease progression [43]. Three years later, the same authors conducted a feasibility study to determine whether HPV E6 mRNA could be detected in the blood of patients with locally advanced CC and to ascertain its relationship with clinical factors and prognosis. In a study of 35 HPV-positive patients, 51.4% exhibited detectable HPV-specific mRNA in their blood, which was associated with primary tumor diameter >4 cm (*p*-value = 0.03) and pelvic lymph node metastasis (*p*-value = 0.03). The presence of circulating HPV E6 mRNA was associated with an elevated risk of recurrence and distant metastasis, indicating that this mRNA may serve as an early marker for metastasis risk [44]. It is known that *BMI-1* is overexpressed in CC and is associated with poor prognosis, but the status of its circulating mRNA was previously unclear. Zhang and colleagues investigated this by detecting circulating *BMI-1* mRNA in CC patients and assessing its diagnostic and prognostic value. Through quantitative reverse transcription polymerase chain reaction (qRT-PCR) analysis of plasma from 109 CC patients, 138 with CIN (cervical intraepithelial neoplasia) and 80 healthy controls, the researchers observed that *BMI-1* mRNA levels were significantly elevated in CC patients, correlating with advanced stage and lymph node metastasis. These findings suggest that *BMI-1* mRNA may serve as a noninvasive marker for CC diagnosis and prognosis.

However, due to the high levels of ribonucleases in the serum of cancer patients, extracellular circulating mRNAs often degrade, and there is a risk of contamination from intracellular mRNAs [45,46]. This has made the use of circulating mRNAs as cancer biomarkers less reliable, limiting their reproducibility and applicability. Consequently, more-dependable RNA-based technologies have been developed, which will be discussed in the following sections.

#### 2.3.2. Non-Coding RNAs

Non-coding RNAs are RNA molecules that do not code for proteins but play crucial roles in the epigenetic regulation of gene expression. This category includes long non-coding RNAs (lncRNAs), short interfering RNAs (siRNAs), microRNAs (miRNAs), and piwi-interacting RNAs (piRNAs), among others. MicroRNAs, small non-coding RNA molecules approximately 19–25 nucleotides in length, regulate post-transcriptional processes in all cells and can act as oncogenes or tumor suppressors in cancer. The expression of miRNAs in cancer patients may exhibit discrepancies in comparison to that observed in healthy controls [47,48]. It seems that the overwhelming majority of precursor miRNA (which are precursors to mature miRNAs) are located in the introns or exons of protein-coding genes (about 70%), while a smaller portion resides in intergenic regions (30%) [49]. In most cases, miRNAs exert their regulatory effects by binding to complementary specific sites at the 3′ end of mRNAs, which may result in either mRNA degradation or suppression of protein synthesis [50]. Mature miRNAs are involved in the regulation of most fundamental biological processes, including cell division, cell cycle phase transitions, apoptosis, cell migration and invasion, angiogenesis, and the formation of the immune response [51,52]. The miRNA database, miRBase (http://www.mirbase.org, last accessed on 1 September 2024), indicates that over 2600 miRNAs have been identified in humans to date, with the number of identified miRNAs growing continuously. It is thought that each miRNA may affect the expression level of more than 100 target genes. It is estimated that approximately 60% of human protein-coding genes are regulated by miRNAs, which suggests that they play a significant role in numerous biological pathways [53]. Aberrant expression of miRNAs contributes to the development of various diseases, including cancer [54]. The first evidence of miRNA involvement in cancer development was provided by Calin and colleagues, who identified a cluster of two miRNAs, miR-15 and miR-16, in the critical 13q14 region frequently deleted in chronic lymphocytic leukemia [55]. Since that time, numerous differentially expressed miRNAs (DEmiRs) have been studied across various types of cancer, including CC.

Progression to intraepithelial precancerous lesions occurs due to multiple disruptions in signaling pathways that regulate the cell cycle, caused by the overexpression of viral proteins E6 and E7, which inactivate the tumor suppressor p53 and pRB1 proteins [56]. This process is multi-staged and may involve a series of genetic and epigenetic changes, including alterations in the transcriptional activity of genes and miRNAs [57]. He et al. published a systematic review focused on the expression profile of microRNAs associated with CC. Their analysis included 3922 CC samples and 2099 controls. The analysis identified 63 DEmiRs between the two groups (42 upregulated and 21 downregulated in CC), with the majority of these microRNAs targeting key oncogenic pathways such as ErbB, MAP kinase, mTOR, p53, TGFβ, and Wnt [58]. Another systematic review [59] explored the potential of microRNAs as markers of progression from cervical intraepithelial neoplasia to CC. Their findings indicated that miR-29a and miR-21 were among the most frequently differentially expressed miRNAs in the context of invasive CC development.

In contrast to the extensive knowledge base surrounding cellular miRNAs and their involvement in cervical carcinogenesis, the understanding of extracellular miRNAs in this context is notably limited, particularly in the context of squamous intraepithelial lesion (SIL) stages. While this is a significant issue for cellular miRNAs, it is even more critical for extracellular miRNAs. Nevertheless, despite the limited number of studies in this area, they collectively contribute to the establishment of an extracellular miRNA signature in SIL and CC. This can assist in understanding the role of miRNAs in cervical carcinogenesis from a global perspective.

To date, we have identified 26 publications focused on studying extracellular microRNAs as diagnostic markers for precancerous and cancerous lesions of the cervix. In a recent study, researchers identified circulating miRNAs associated with CC and CIN to create a non-invasive classifier for cervical lesions. The study comprised five stages: miRNA screening, validation, classifier development, independent validation, and in silico analysis. Six miRNAs (miR-26b-5p, miR-146b-5p, miR-191-5p, miR-484, miR-574-3p, and miR-625-3p) were identified and used to develop a classifier with an accuracy of 0.7218 in validation samples and 0.7021 in an independent cohort. These findings suggest that these miRNAs could serve as non-invasive biomarkers for cervical lesions [60].

The majority of studies were focused on extracellular microRNAs present in patient’s blood. However, there has been a paucity of research on microRNAs in cervical mucus in the context of HPV-associated cervical pathology. Kotani et al. [61] investigated whether miRNA expression in cervical mucus could improve the detection of cervical neoplasia. In 709 patients, a combination of five miRNAs (miR-126-3p, miR-451a, miR-144-3p, miR-20b-5p, miR-155-5p) was found to be highly accurate for cancer detection with an AUC (area under curve) of 0.956. A nomogram incorporating these miRNAs, HPV genotype, and age showed strong predictive power and consistency, suggesting its potential as an additional screening tool for cervical neoplasia.

### 2.4. Extracellular Vesicle (EV) miRNA

#### 2.4.1. EV Biogenesis, Composition, and Characterization

As previously noted, miRNAs can be found both within cellular compartments and in the extracellular space, associated with proteins, lipids, or within EVs [62]. The presence of microRNAs in blood serum was first reported in 2008 by Lawrie et al., who noted elevated expression levels of miR-21 in the serum of patients with diffuse large B-cell lymphoma [63]. Since then, numerous studies have demonstrated the presence of extracellular microRNAs in various biological fluids, including plasma, saliva, urine, and cerebrospinal fluid [64]. Unlike cellular microRNAs, which are rapidly degraded in the RNase-rich extracellular environment, extracellular microRNAs exhibit good stability and can withstand boiling, extreme pH, long-term storage, and freeze–thaw cycles [65]. Two main mechanisms have been proposed for protecting extracellular miRNAs from ribonuclease degradation. One involves the conjugation of miRNAs with Argonaute (Ago) proteins, particularly Ago2. The miRNA/Ago2 complexes are apparently non-specific residues that arise from physiological cell activity and cell death (apoptosis or necrosis) [66]. The other protective mechanism is the encapsulation of miRNAs within EVs. In this case, EV miRNAs can be transported to recipient cells, altering the expression of protein-coding genes and thereby regulating various signaling pathways in the cell [67,68]. The incorporation of miRNAs into EVs is not a random event, and the mechanisms underlying this process are not yet fully understood [69]. Several regulatory mechanisms have been described through which cells can selectively control the entry of microRNAs into EVs. MiRNA sorting can be facilitated by RNA-binding proteins such as hnRNPA2B1, SYNCRIP, Ago2, YBX-1, MEX3C, MVP, and La protein, which selectively bind to miRNAs and transport them into vesicles [70]. The hnRNPs are RNA binding proteins and they complex with heterogeneous nuclear RNA (hnRNA). For example, sumoylated hnRNPA2B1 recognizes GGAG/UGCA motifs at the 3′ end of miRNAs, binds to them, and ensures the selection of specific miRNAs into EVs [71]. SYNCRIP, another member of the hnRNP-Q protein family, participates in loading specific microRNAs into EVs. A deficiency of this protein leads to the accumulation of microRNAs in the cytosol [72]. MVP is a ribonucleoprotein involved in transporting RNA from the nucleus to the cytoplasm [73]. Recent evidence suggests that MVP may play a role in sorting EV miRNAs. MVP knockout in CT26 colon cancer cells resulted in an increase in cellular miR-193a but a decrease in its levels in EVs derived from CT26 [74]. Additionally, some membrane proteins involved in vesicle biogenesis, such as Cav-1, nSMase2, and Vps4A, selectively transfer miRNAs into EVs [70]. nSMase2 is a hydrolase involved in the metabolism of sphingolipids, which are crucial components of the plasma membrane. Overexpression of this protein leads to an increase in the amount of miRNAs (specifically, miR-16 and miR-146a) in EVs, while a reduction in its expression has the opposite effect [75,76]. A sorting mechanism associated with the 3′-end of miRNAs has also been described. Post-transcriptional modification of miRNAs, especially the uridylation of the 3′-end, enhances the sorting of ribonucleic acids into EVs, whereas adenylation of the 3′-end is associated with the opposite effect [77]. Pathological conditions such as chronic lung diseases, immune responses, neuroinflammation, diabetes, cancer, and cardiovascular diseases have been demonstrated to result in dysregulation of EV miRNAs [70].

**Figure 2:**
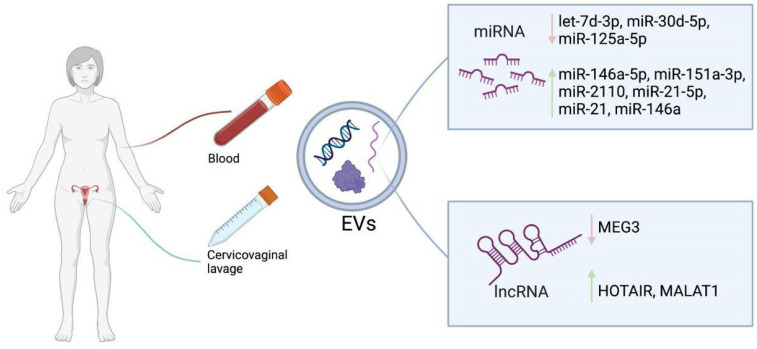
Illustration depicting liquid biopsy where blood or cervicovaginal lavage is obtained from a patient to isolate and characterize EVs, miRNA, and/or IncRNA for eventual diagnostics. EVs—extracellular vesicles.

Exosomes are a subclass of small membranous EVs released by both tumor and non-tumor cells into the body fluids or extracellular environment. These vesicles play a pivotal role in cellular communication, facilitating the transfer of information from donor cells to recipient cells [78]. The biogenesis of exosomes is a complex process involving the invagination of the plasma membrane, the formation of multivesicular bodies (MVBs), and the secretion of exosomes. Multivesicular bodies (MVBs) are endocytic structures that are formed by the inward budding of endosomal membranes. Vesicles that accumulate within MVBs, known as intraluminal vesicles, are released as exosomes when MVBs fuse with the plasma membrane [79]. The lumen of exosomes contains biologically active molecules from donor cells, including DNA, RNA, lipids, and proteins, highlighting the crucial role of exosomes in genetic information exchange [80]. Exosomes and other EVs that are formed and released by cells into extracellular space through different pathways employ autocrine and paracrine signaling to regulate a multitude of cellular processes and to modulate their microenvironment [81,82]. They can modify the biological phenotype of recipient cells by regulating RNA expression and stimulating receptor activation. Cancer cells exchange EVs with stromal cells, thereby creating a microenvironment that enhances tumor invasion and proliferation [83]. A growing body of evidence suggests that proteins, nucleic acids, and other molecules are integrated into exosomes and expressed differentially in tumors of various origins. A growing body of evidence suggests that proteins, nucleic acids, and other molecules are integrated into EVs and expressed differentially in tumors of various origins. EVs are protected from degradation by their lipid bilayer membranes, which contribute to their stability in biological fluids, even in harsh tumor microenvironments. This significant biological stability enables the long-term preservation of samples for the isolation and detection of EVs [84]. Considering that EVs are released by living cells, their distinctive content can mirror the pathophysiological condition of the parent cell, thereby serving as valuable biomarkers for dynamically tracking disease progression [82]. It has been suggested that the amount of EVs secreted may be correlated with tumor invasiveness, both in vitro and in vivo. It is believed that these EVs contribute to increased migration and proliferation of tumor cells, which may lead to metastasis [83]. Overall, the cargo, sorted into EVs, particularly microRNAs, may serve as promising biomarkers for diagnosis, treatment monitoring, and prognosis in cancer patients, opening new possibilities for liquid biopsy (Figure 2). One challenge in utilizing EVs for liquid biopsy is the absence of standardized protocols for their isolation and identification, which affects the purity and quality of the molecules obtained [85,86]. Tumor-derived EVs represent a minor proportion of all EVs present in different biological fluids, underscoring the importance of developing and implementing sensitive detection methods for EV-based diagnostics. Currently, numerous methods have been developed for isolating EVs and detecting EV proteins and nucleic acids [87,88,89]. Nevertheless, further research is necessary to develop an accessible platform that is both highly sensitive and specific for EV separation and detection.

#### 2.4.2. EV-miRNA for CC Diagnosis

The objective of one study was to ascertain whether the intracellular and extracellular miRNA profiles of EVs secreted by HPV-positive cancer cells are influenced by the expression of E6/E7 oncogenes. A distinctive signature consisting of seven miRNAs was identified among the most abundant miRNAs in EVs released by HeLa cells. Upon E6/E7 silencing, a notable downregulation of let-7d-5p, miR-20a-5p, miR-378a-3p, miR-423-3p, miR-7-5p, and miR-92a-3p was observed, while miR-21-5p exhibited a significant upregulation. E6/E7 silencing affected key miRNAs involved in cell proliferation, senescence, and apoptosis, both within cells and in EVs. These findings suggest that E6/E7-regulated miRNAs play a significant role in the growth and survival of HPV-positive cancer cells [90]. EVs can be released by precancerous and cancerous cervical cells and are present in abundance in vaginal lavage fluid and plasma, playing a vital role in the different stages of CC formation and development.

There is significant interest in studying EV miRNAs in the blood plasma of patients with precancerous and cancerous lesions of the cervix. Ma et al. [91] analyzed plasma samples from 97 CC patients and 87 healthy women to assess the diagnostic potential of various miRNAs. The researchers identified the upregulation of four EV miRNAs (miR-146a-5p, miR-151a-3p, miR-2110, and miR-21-5p). Additionally, the study examined miRNAs in biopsies and found elevated levels of miR-146a-5p and miR-21-5p in CC tissue samples, with miR-146a-5p, miR-151a-3p, and miR-2110 increased in plasma EVs. These findings indicate that a significant proportion of circulating microRNAs originate from tumor tissues. Zheng et al. [92] conducted sequencing of EV miRNAs in 121 plasma samples from 23 healthy women, 5 patients with CIN I, 59 with CIN II-III, 21 with squamous cell carcinoma of the cervix (SCC), and 13 with adenocarcinoma (ACC). At this stage, 312 miRNAs were identified. The CIN I- samples were used as reference data for comparison with the other sample groups (CIN II-III, SCC, and ACC). The comparative analysis revealed 37 DEmiRs. A subgroup of miRNAs from these 37 DEmiRs was then selected using the random forest algorithm. This led to the identification of an optimal panel of eight miRNAs (let-7a-3p, let-7d-3p, miR-30d-5p, miR-144-5p, miR-182-5p, miR-183-5p, miR-215-5p, and miR-4443), with potential to be used in clinical diagnostics. This study represents one of the most comprehensive investigations into miRNA analysis in the context of CC. It has undergone several phases, during which miR-30d-5p and let-7d-3p have been identified as valuable diagnostic biomarkers for non-invasive screening of CC and its precursors (Figure 1, Table 1). Lv and colleagues [93] investigated the potential of EV miRNAs as biomarkers for CC diagnosis. EV RNA-sequencing of samples from six CC patients and six healthy controls identified 39 differentially expressed miRNAs. Further analysis of plasma EV miR-125a-5p in 60 subjects revealed significantly lower levels in CC patients compared to controls. ROC (receiver operating characteristic) curve analysis indicated that miR-125a-5p could be a potential diagnostic marker, with a sensitivity of 59.1% and specificity of 84.2%. However, more samples are needed to confirm these findings.

Cervicovaginal lavage fluid (CVF) is another type of biofluid that was shown to contain EVs and be used for CC screening. Lavage fluid, sterile saline or phosphate-buffered saline, which usually ranges 2.5–10 mL in volume, is used for rinsing lower reproductive tract and collecting mucosal surface consisting of host and microbial cells, vesicles, proteins, peptides, metabolites, and other components [94]. Compared to serum and plasma, CVF contains fewer contaminants such as proteins which simplifies EV isolation and allows for the obtaining of EV samples with high purity [95,96]. Such biofluid is easy to collect, which makes it applicable for screening especially at point-of-need. However, variability of collected CVF volume can lead to order of magnitude differences in end-results. Solutions to this drawback have already been presented, showing that CVF can potentially become the most optimal biofluid for analysis [94]. In CC patients, EV levels of miR-21, miR-146a [97], and lncRNAs HOTAIR and MALAT1 were elevated in cervicovaginal lavage samples, while lncRNA MEG3 was decreased [98].

Table 1 includes extracellular miRNAs identified in EVs, expression levels of which were found to be either increased or decreased in CC studies. The number of research studies that evaluated EV miRNA as CC diagnostic markers is still sparse and used either ExoQuick (System Biosciences, Palo Alto, CA, USA), exoEasy Maxi kit (Qiagen, Germantown, MD, USA), or differential centrifugation for EV isolation. ExoQuick is an EV isolation method based on polyethylene glycol polymer (PEG)-based precipitation, while the exoEasy Maxi kit utilizes affinity spin columns for EV purification. Differential centrifugation is the oldest method for EV isolation, still considered to be a gold standard, where biofluid components are separated by performing consecutive steps with specific centrifugation speeds to first remove contaminants and finally pellet small EVs at ≈100,000× *g*. Numerous methods for EV isolation exist [99]; however, since the chosen principle and protocol can lead to the isolation of only specific EV subpopulations that can be enriched with particular types of cargo, it is difficult to compare the presented miRNA biomarkers. Another factor that can impact miRNA results is RNA isolation. TRIzol (ThermoFisher, Waltham, MA, USA), ISOGEN-LS (Fujifilm, Tokyo, Japan), miRNeasy Serum/Plasma (Qiagen, Germantown, MD, USA), RNeasy Micro (Qiagen, Germantown, MD, USA), QIAzol Lysis Reagent (Qiagen, Germantown, MD, USA), MirVana miRNA isolation (ThermoFisher, Waltham, MA, USA), RNAzol RT (Sigma-Aldrich, St. Louis, MO, USA), and Total RNA Isolation (QuantoBio, Beijing, China) kits contain acidified phenol solutions, which, after the introduction of chloroform, allow for the phase separation of biofluid into an aqueous phase containing RNA and interphase, with the organic phase containing DNA, proteins, and lipids. MirVana miRNA, miRNeasy Serum/Plasma, and RNeasy Micro kits allow for the purification of RNA via its capturing and washing on spin columns containing special filters (e.g., glass fiber, silica membrane). MiRNeasy Serum/Plasma Advanced kit (Qiagen, Germantown, MD, USA) is a phenol-free alternative to miRNeasy Serum/Plasma. MiRCURY RNA isolation kit (Qiagen, Germantown, MD, USA) is another approach that is based on spin column chromatography, avoiding the use of phenol or chloroform for RNA capture. Since the listed RNA isolation methods can provide different purity, yield, and RNA fractions, it can be an additional factor leading to only a small overlap in the presented CC markers.

**Table 1:** Expression profile of extracellular miRNAs in human precancerous and cancerous lesions of the cervix obtained after isolation from biofluids or EVs using different methods and subsequent PCR.

Sample Type	EV Isolation	RNA Isolation	Increased Expression	Decreased Expression	Ref.
miRNAs in EVs					
Tissues, plasma, plasma EVs	ExoQuick	mirVana Paris (plasma, EVs) TRIzol (tissues)	miR-146a-5p miR-151a-3p miR-2110 miR-21-5p	-	[91]
Tissues, plasma EVs	ExoQuick with RNase A	miRNeasy Micro (EVs) TRIzol (tissues)	-	let-7d-3p miR-30d-5p	[92]
Plasma, plasma EVs	exoEasy Maxi kit	miRNeasy (EVs); QIAzol Lysis Reagent (plasma)	-	miR-125a-5p	[93]
Cervicovaginal lavage fluid	Differential centrifugation	mirVana miRNA isolation	miR-21 miR-146a	-	[97]
Extracellular miRNAs					
Serum	-	ISOGEN-LS	miR-1290	-	[100]
Serum	-	miRNeasy Serum/Plasma	miR-196a	-	[101]
Serum	-	Total RNA Isolation	miR-16-2 miR-497	miR-195 miR-2861	[102]
Tissues, serum	-	miRNeasy Mini	miR-425-5p	-	[103]
Tissues, serum, cells (HeLa, SiHa)	-	TRIzol	miR-1266	-	[104]
Tissues, serum	-	mirVana miRNA Isolation	miR-9 miR-192 miR-205	-	[105]
Serum	-	miRNeasy Serum/Plasma	miR-21	miR-125b miR-370	[106]
Tissues, plasma	-	Acidified phenol, chloroform (plasma); miRCURY RNA isolation (tissue)	miR-21	miR-214 miR-34a miR-200a	[107]
Whole blood, serum	-	RNAzol LS	miR-152	-	[108]
Tissues, serum	-	TRIzol	miR-15b	miR-34a miR-218	[109]
Plasma	-	miRNeasy Serum/Plasma	miR-26b-5p miR-146b-5p miR-191-5p miR-484 miR-574-3p miR-625-3p	-	[60]
Plasma	-	miRVana PARIS	-	miR-145	[110]
Serum	-	miRNeasy Serum/Plasma Advanced	-	miR-638 miR-521 miR-1914-5p miR-203a-3p	[111]
Tissues, serum	-	ISOGEN-LS (serum); RNeasy Micro (tissues)	-	miR-100	[112]
Serum	-	miRNeasy	miR-9 miR-10a miR-20a miR-196a	-	[113]
Serum	-	mirVana PARIS	miR-646 miR-141 miR-542-3p	-	[114]
Serum	-	TRIzol	miR-21 miR-29a miR-25 miR-200a miR-486-5p	-	[115]
Serum	-	mirVana	-	miR-101-3p	[116]
Plasma	-	QIAamp circulating Nucleic Acid	miR-127 miR-205	-	[117]
Tissues, serum	-	TRIzol (tissue) miRNeasy Serum/Plasma (serum)	miR-205	-	[118]
Tissues, serum	-	TRIzol (total RNA) mirVana (serum miRNA)	-	miR-218	[119]
Tissues, serum	-	miRNeasy Mini	miR-486-5p		[120]
Mucus, tissue	-	miRNeasy Mini (mucus) TRIzol (tissue)	miR-126-3p miR-20b-5p miR-451a miR-144-3p	-	[121]
Mucus	-	miRNeasy Mini	miR-126-3p miR-451a miR-144-3p miR-20b-5p miR-155-5p	-	[61]
Serum	-	miRNA extraction	miR-92a	-	[122]
Serum	-	TRIzol (total RNA) mirVana (serum miRNA)	-	miR-218	[123]

#### 2.4.3. Gene-Targets of EV-miRNA Associated with CC

**Figure 3:**
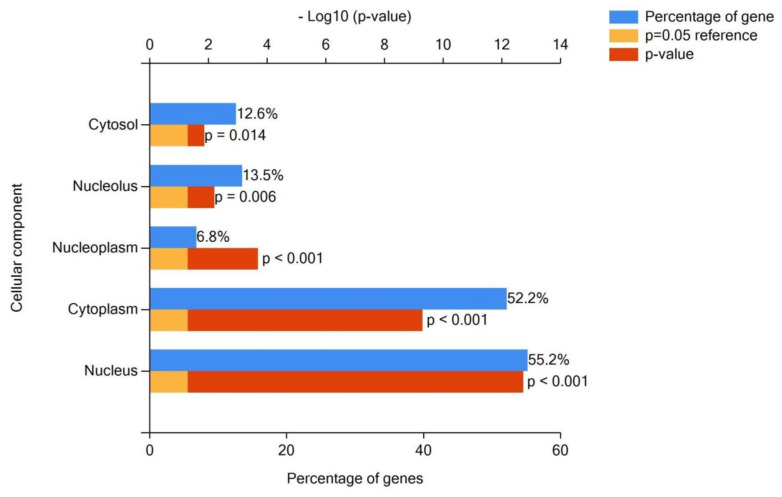
Cellular localization of experimentally validated gene-targets protein products of CC microRNA markers presented in Table 1 and Table 2, generated by the FunRich (Version 3.1, last accessed on 1 September 2024) program.

**Figure 4:**
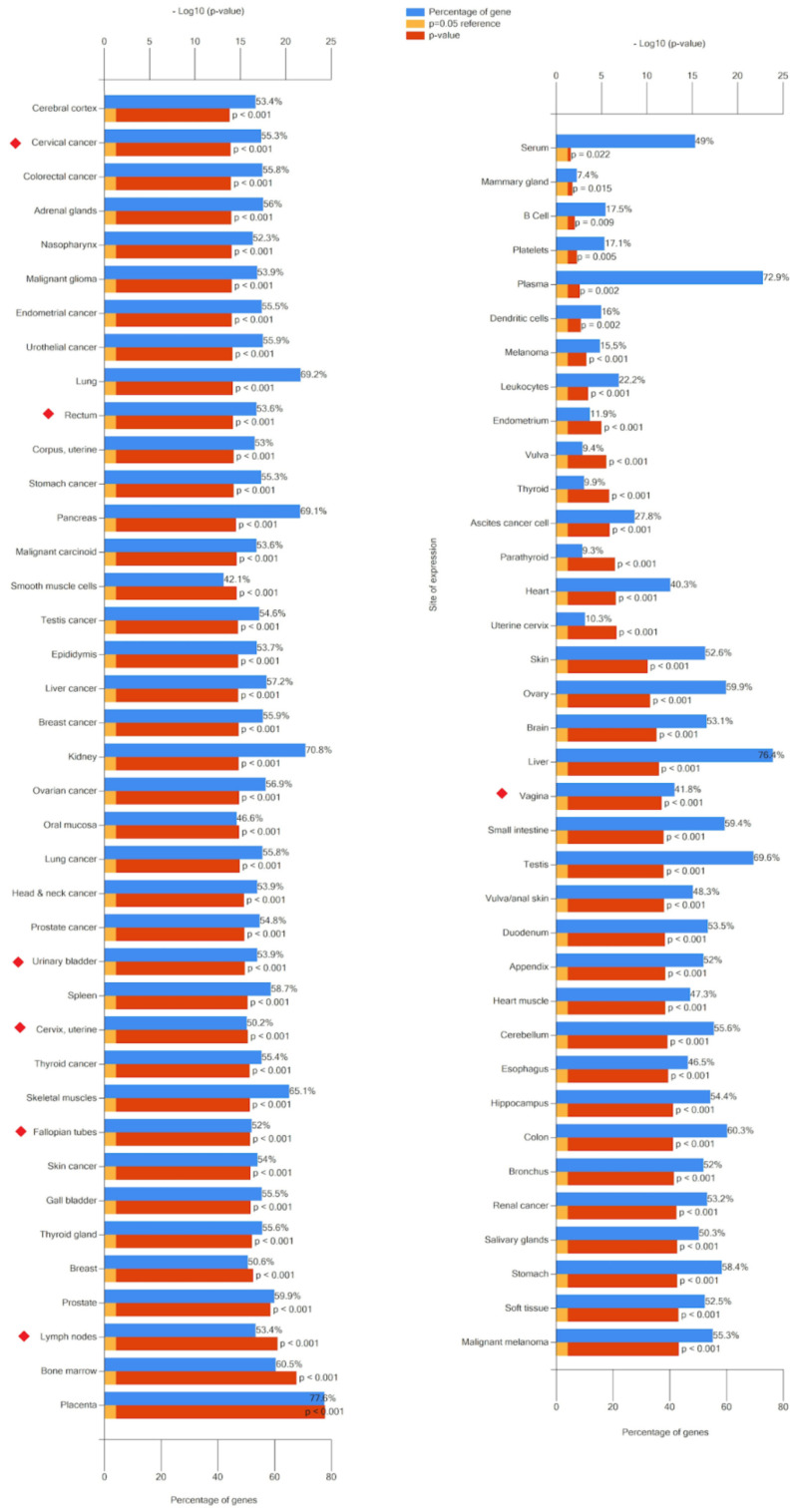
Organ expression of target genes for CC miRNA markers. Data are generated using the FunRich (Version 3.1, last accessed on 1 September 2024) tool. The red diamonds indicate the main sites of metastasis for the CC.

**Figure 5:**
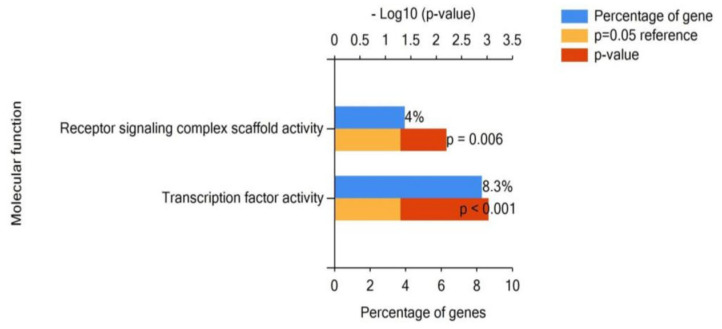
Molecular function of gene-targets protein products for CC miRNA markers. Data are generated using the FunRich (Version 3.1, last accessed on 1 September 2024) tool.

**Figure 6:**
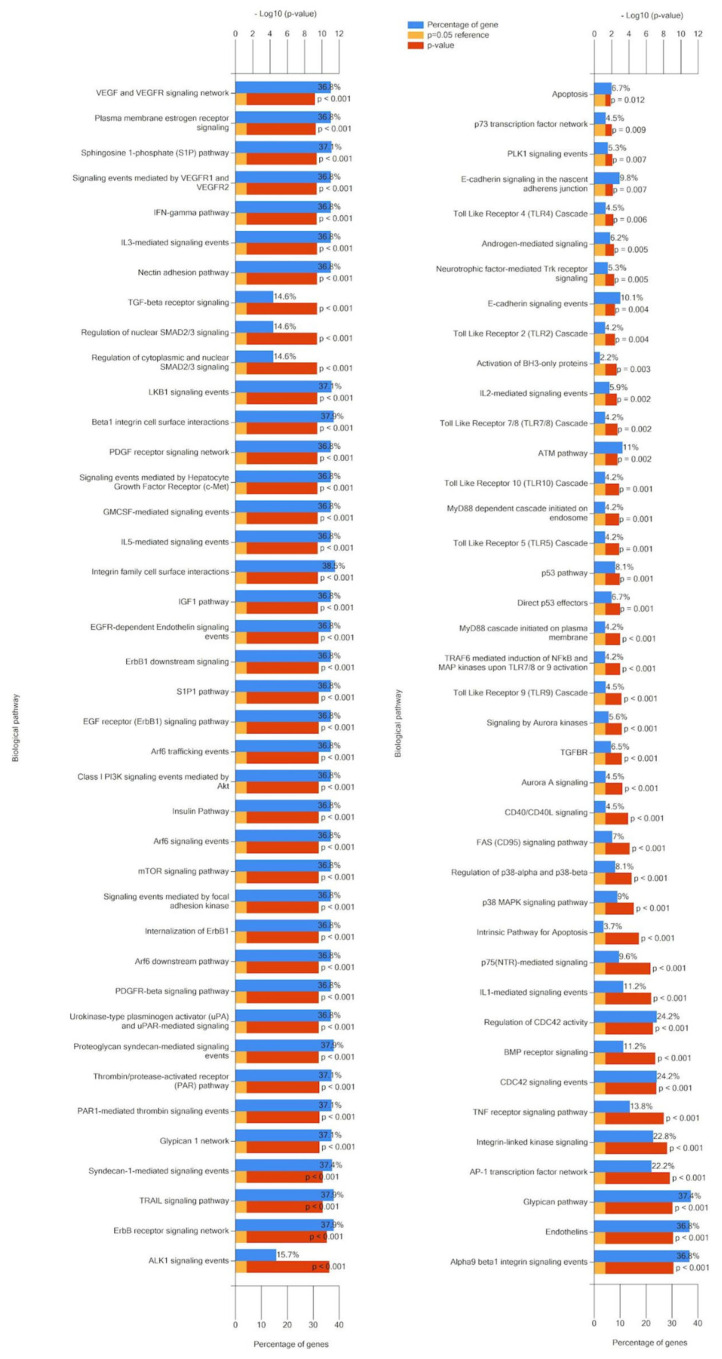
Signaling pathways formed by gene-targets protein products for CC miRNA markers. Data are generated by using the FunRich (Version 3.1, last accessed on 1 September 2024) tool.

Functional enrichment of the gene set, namely, experimentally confirmed gene-targets for 28 (hsa-miR-370-3p, -miR-646, -miR-484, -miR-638, -miR-2861, -miR-21-5p, -miR-214-3p, -miR-191 -5p, -miR-151a-3p, -miR-1266-5p, -miR-101-3p, -miR-9-5p, -miR-542-3p, -miR-486-5p, -miR-30d- 5p, -miR-2110, -miR-200a-3p, -miR-1914-5p, -miR-146a-5p, -miR-125a-5p, -miR-205-5p, -miR-203a-3p, -miR-192-5p, -miR-1290, -miR-146b-5p, -miR-26b-5p, -miR-521, -miR-16-2-3p) of 49 miRNAs, associated with CC (Table 1 and Table 2), were identified using the open-source platform miRWalk (Table S1). The FunRich (Version 3.1, last accessed on 1 September 2024) program plotted cellular localization (Figure 3), expression site (Figure 4), molecular function (Figure 5), and involvement in signaling pathways of the protein products of these target genes (Figure 6) using gene enrichment analyses. A *p*-value of <0.05 was considered significant. Accordingly, this program performed functional enrichment of these genes to analyze the localization and function of their protein products and presented the results in the form of graphs in which the blue box is for a percentage of genes, the yellow box is for reference *p*-value, equal to 0.05, and the red box reflects the determined *p*-values.

Figure 4 shows that the target genes of CC miRNA markers are expressed in many organs, including the cervix itself, vagina, bladder, ureter, rectum, and uterine appendages, which are the main sites of metastasis for this type of neoplasia. Their significant molecular functions were such as receptor signaling complex scaffold activity and transcription factor activity (Figure 5). The 55.2% and 52.2% of the cellular component of gene-targets were the nucleus (*p*-value < 0.001) and cytoplasm, respectively (Figure 3). In a recent review [124], the effects of HPV proteins on the PI3K/Akt, Wnt/β-catenin, ERK/MAPK, NF-κB, YY1, AP-1, JAK/STAT, and CXCL12/CXCR4 signaling pathways in the host cells were discussed in light of the progression of CC. These data are in accordance with the gene enrichment analysis of target genes (Figure 6), which form such signaling pathways under the control of miRNA markers of CC. Among them is PI3K/mTORC2/AKT signaling activated by HPV oncoproteins (E6 and E7) to delay or prevent apoptosis, providing virus replication. This state is maintained due to the direct inhibitory effect of E6 on the p53 protein through its ubiquitination, which may be the cause of oncotransformation of the host cell. It follows from Figure 6 that most of the target genes are enriched in proteoglycan syndecan-mediated signaling, the glypican pathway, the Erb-B receptor signaling network, urokinase-type plasminogen activator signaling, and the VEGF and VEGFR signaling networks, which play a role in cancer cell proliferation, invasion, and metastasis.

In contrast to the extensive knowledge base surrounding cellular miRNAs and their involvement in cervical carcinogenesis, the understanding of extracellular miRNAs in this context is notably limited, particularly in the squamous intraepithelial lesion (SIL) stages. While this is a significant issue for cellular miRNAs, it is even more critical for extracellular miRNAs. Nevertheless, despite the limited number of studies in this area, they collectively contribute to the establishment of an extracellular miRNA signature in SIL and CC. This can assist in improving our understanding of the role of miRNAs in cervical carcinogenesis from a global perspective.

## 3. Conclusions

CC is distinguished by its high potential for prevention and treatment among all types of cancer [125]. Despite this, it continues to be a serious public health issue due to its high morbidity and mortality rates, especially among women of reproductive age (30 to 50 years old) [126]. CC has a favorable prognosis in case of early diagnosis. However, existing screening methods (oncocytology, HPV testing) have significant limitations [102], necessitating the search for new biomarkers. LB, which involves the analysis of biofluids that can be easily obtained from patients, may offer a promising non-invasive approach for the early and differential diagnosis of precancerous and cancerous cervical conditions. While numerous oncological markers have been identified in patient blood (e.g., CEA (Carcinoembryonic antigen), SCC-Ag), their specificity and sensitivity for CC remain limited. In addition to CTC and free DNA analysis, which have their own advantages and drawbacks, miRNA is another type of liquid biopsy that has received attention due to its higher quantity in biological fluids, its protection against degradation, its potential for attaining high sensitivity and specificity, and its ability to reflect miRNA expression in tumor cells. miRNA biopsies also allow us to determine the malignancy grade, to detect disease progression, and to monitor therapy response. The target genes of CC miRNA markers are expressed in various organs, including the cervix, vagina, bladder, ureter, rectum, and uterine appendages. They play a role in receptor signaling complex scaffold activity and transcription factor activity. The understanding of extracellular miRNAs in cervical carcinogenesis is limited, but their collective contribution can help us to understand their global role.

Despite the considerable advantages of EV miRNAs in cancer diagnostics, several challenges remain to be addressed. First, biological fluids are a rich source of EVs from various origins, which can complicate the isolation of tumor-derived EVs. Secondly, although numerous EV isolation methods have been proposed, the purity and quality of the isolated EVs vary depending on the method employed. Furthermore, there is still no standard protocol for EV isolation and detection. A third issue to be considered is the lack of established endogenous miRNA controls for normalizing miRNA levels. Finally, numerous studies on EV miRNAs have been conducted on small sample sizes, whereas larger-scale studies are necessary for accurate data comparison and validation. Further investigation of EV miRNAs in blood may yield new insights into non-invasive cancer diagnostics in the near future.

## Figures and Tables

**Table 2:** ROC analysis results from different studies to evaluate the capabilities of chosen markers to differentiate between different groups of patients and normal controls. Studies show that miRNA markers have a high potential in diagnostics and treatment monitoring. To improve the performance of the diagnostic approach, a research group also combined miRNA markers with squamous cell carcinoma-related antigen (SCC-Ag) and demonstrated the positive effect of the combination on their ability to differentiate between cervical squamous cell carcinoma patients and healthy controls in comparison with miRNA markers and SCC-Ag alone [111]. Coupling existing methods with miRNA and potentially other biomarkers is a route that can significantly improve our ability to diagnose in the early stages, and furthermore to identify the subtypes of diseases.

miRNA	AUC	Se (%)	Sp (%)	Ref.
**miRNAs in EVs**				
miR-146a-5p; miR-151a-3p; miR-2110; miR-21-5p Panel (combined) Panels (training; testing; external validation)	0.77; 0.752; 0.721; 0.677 0.855 0.911; 0.774; 0.786	- - - -	- - - -	[91]
EV let-7d-3p; EV miR-30d-5p EV panel (sequencing): let-7d-3p, miR-30d-5p EV panel (ddPCR): let-7d-3p, miR-30d-5p	0.822; 0.790 0.922 0.828	- - -	- - -	[92]
EV miR-125a-5p	0.713	-	-	[93]
miR-21 miR-146a	-	-	-	[97]
**Extracellular miRNAs**				
miR-1290	-	90.3	62.2	[100]
miR-196a	-	-	-	[101]
Panel: miR-16-2, miR-497, miR-195, miR-2861 (CC vs. N; CC vs. CIN; CIN vs. N; CC (Stage I) vs. N; CC (Stage I and II) vs. N)	0.849; 0.829; 0.734; 0.863; 0.873	73.1; 71.4; 62.6; NA; NA	88.4; 67.2; 88.9; NA; NA	[102]
miR-425-5p				[103]
miR-1266				[104]
miR-9 (CC vs. N; CC vs. PCC; PCC vs. N) miR-192 (CC vs. N; CC vs. PCC; PCC vs. N) miR-205 (CC vs. N; CC vs. PCC; PCC vs. N)	0.80; 0.71; 0.59 0.87; 0.85; 0.59 0.79; 0.71; 0.64	100; 52.9; 77.8 100; 58.8; 83.3 88.2; 35.3; 66.7	94.4; 94.4; 94.4 94.4; 83.3; 94.4 88.9; 94.4; 88.9	[105]
miR-21 (CC vs. N; CC vs. CIN) miR-125b (CC vs. N; CC vs. CIN) miR-370 (CC vs. N; CC vs. CIN) Panel: miR-21, miR-125b, miR-370 (CC vs. N; CC vs. CIN)	0.783; 0.689 0.642; 0.735 0.822; 0.821 0.912; 0.897	-	-	[89]
miR-21 (CIN1- vs. CIN2+) miR-34a (CIN1- vs. CIN2+) miR-200a (CIN1- vs. CIN2+) miR-214 (CIN1- vs. CIN2+)	0.613 0.508 0.615 0.505	-	-	[90]
miR-152 (CIN vs. N; CC vs. N)	0.831; 0.935	-	-	[108]
miR-34a (LSIL vs. N; SCC vs. N) miR-15b (LSIL vs. N; SCC vs. N) miR-218 (LSIL vs. N; SCC vs. N)	0.995; 0.951 0.677; 0.817 0.987; 0.842	-	-	[109]
Panel: miR-26b-5p, miR-146b-5p, miR-191-5p, miR-484, miR-574-3p, miR-625-3p (CC/CIN vs. N, independent validation)	0.690	80.8	57.1	[60]
miR-145 (CC vs. N; CC vs. CIN; CR vs. IR)	0.848; 0.828; 0.801	81.7; 91.7; 64.7	63.3; 54.2; 84.6	[110]
miR-638 (CSCC vs. CIN; CSCC vs. N) miR-521 (CSCC vs. CIN; CSCC vs. N) miR-1914-5p (CSCC vs. CIN; CSCC vs. N) miR-203a-3p (CSCC vs. CIN; CSCC vs. N) Panel: miR-521, SCC-Ag (CSCC vs. N) Panel: miR-638, SCC-Ag (CSCC vs. N) Panel: miR-638, miR-521, SCC-Ag (CSCC vs. N) SCC-Ag without miR (CSCC vs. N)	0.681; 0.734 0.716; 0.742 0.717; 0.626 0.651; 0.660 0.904 0.956 0.958 0.872	85.0; 80.0 62.5; 80.0 82.5; 85.0 42.5; 75.0 - - - 74.4	46.2; 61.0 76.9; 65.9 61.5; 43.9 82.1; 53.7 - - - 92.7	[111]
miR-100	0.879	-	-	[112]
Panel: miR-9, miR-10a, miR-20a, miR-196a (CIN vs. N)	0.886	-	-	[113]
miR-646 miR-141 miR-542-3p	-	-	-	[114]
miR-21 (CC vs. N) miR-29a (CC vs. N) miR-25 (CC vs. N) miR-200a (CC vs. N) miR-486-5p (CC vs. N) Panel: miR-21; miR-29a; miR-25; miR-200a; miR-486-5p (CC vs. N)	0.819 0.819 0.726 0.658 0.685 0.908	- - - - - 81.0	- - - - - 88.6	[115]
miR-101-3p (CC vs. N)	-	-	-	[116]
miR-127 (CC vs. N) miR-205 (CC vs. N)	0.82 0.84	75.5 72.0	83.3 82.3	[117]
miR-205 (CC stage Ib-IIa vs. CC stage IIb,IIIa) miR-205 (CC vs. N)	0.74 0.69	76.5 71.1	73.1 72.7	[118]
miR-218 (CC vs. N)	-	-	-	[119]
miR-486-5p (CC vs. N)	0.90	-	-	[120]
miR-126-3p (CC vs. N; CC+AD vs. N) miR-20b-5p (CC vs. N; CC+AD vs. N) miR-451a (CC vs. N; CC+AD vs. N) miR-144-3p (CC vs. N; CC+AD vs. N)	0.91; 0.89 0.94; 0.90 0.96; 0.94 0.95; 0.93	86; 81 83; 74 91; 83 89; 87	91; 91 93; 93 95; 91 93; 89	[121]
miR-126-3p (CC vs. N; CC+AD vs. N) miR-20b-5p (CC vs. N; CC+AD vs. N) miR-451a (CC vs. N; CC+AD vs. N) miR-144-3p (CC vs. N; CC+AD vs. N)	0.91; 0.89 0.94; 0.90 0.96; 0.94 0.95; 0.93	86; 81 83; 74 91; 83 89; 87	91; 91 93; 93 95; 91 93; 89	[61]
mir-92a	0.83	69.6	80.4	[122]
miR-218	-	-	-	[123]

## Supplementary Materials

The following supporting information can be downloaded at: https://www.mdpi.com/article/10.3390/ijms252413271/s1.

## References

[1] Viveros-Carreño D., Fernandes A., Pareja R. (2023). Updates on cervical cancer prevention. Int. J. Gynecol. Cancer.

[2] Singh D., Vignat J., Lorenzoni V., Eslahi M., Ginsburg O., Lauby-Secretan B., Arbyn M., Basu P., Bray F., Vaccarella S. (2023). Global estimates of incidence and mortality of cervical cancer in 2020: A baseline analysis of the WHO Global Cervical Cancer Elimination Initiative. Lancet Glob. Health.

[3] Chesson H.W., Dunne E.F., Hariri S., Markowitz L.E. (2014). The Estimated Lifetime Probability of Acquiring Human Papillomavirus in the United States. Sex. Transm. Dis..

[4] Gates A., Pillay J., Reynolds D., Stirling R., Traversy G., Korownyk C., Moore A., Thériault G., Thombs B.D., Little J. (2021). Screening for the prevention and early detection of cervical cancer: Protocol for systematic reviews to inform Canadian recommendations. Syst. Rev..

[5] World Health Organization (2020). Global Strategy to Accelerate the Elimination of Cervical Cancer as a Public Health Problem.

[6] Weller D., Vedsted P., Rubin G., Walter F.M., Emery J., Scott S., Campbell C., Andersen R.S., Hamilton W., Olesen F. (2012). The Aarhus statement: Improving design and reporting of studies on early cancer diagnosis. Br. J. Cancer.

[7] Pesola F., Sasieni P. (2019). Impact of screening on cervical cancer incidence in England: A time trend analysis. BMJ Open.

[8] van der Aa M.A., Pukkala E., Coebergh J.W.W., Anttila A., Siesling S. (2008). Mass screening programmes and trends in cervical cancer in Finland and The Netherlands. Int. J. Cancer.

[9] Vaccarella S., Lortet-Tieulent J., Plummer M., Franceschi S., Bray F. (2013). Worldwide trends in cervical cancer incidence: Impact of screening against changes in disease risk factors. Eur. J. Cancer.

[10] Liang L.A., Einzmann T., Franzen A., Schwarzer K., Schauberger G., Schriefer D., Radde K., Zeissig S.R., Ikenberg H., Meijer C.J.L.M. (2021). Cervical Cancer Screening: Comparison of Conventional Pap Smear Test, Liquid-Based Cytology, and Human Papillomavirus Testing as Stand-alone or Cotesting Strategies. Cancer Epidemiol. Biomark. Prev..

[11] Nanda K., McCrory D.C., Myers E.R., Bastian L.A., Hasselblad V., Hickey J.D., Matchar D.B. (2000). Accuracy of the Papanicolaou Test in Screening for and Follow-up of Cervical Cytologic Abnormalities. Ann. Intern. Med..

[12] Kurtycz D.F.I., Staats P.N., Chute D.J., Russell D., Pavelec D., Monaco S.E., Friedlander M.A., Wilbur D.C., Nayar R. (2017). Bethesda Interobserver Reproducibility Study-2 (BIRST-2): Bethesda System 2014. J. Am. Soc. Cytopathol..

[13] Gupta R., Hariprasad R., Dhanasekaran K., Sodhani P., Mehrotra R., Kumar N., Gupta S. (2020). Reappraisal of cytology-histology correlation in cervical cytology based on the recent American Society of Cytopathology guidelines (2017) at a cancer research centre. Cytopathology.

[14] Austin R.M., Ramzy I. (1998). Increased detection of epithelial cell abnormalities by liquid-based gynecologic cytology preparations. A review of accumulated data. Acta Cytol..

[15] Klinkhamer P.J.J.M., Meerding W.J., Rosier P.F.W.M., Hanselaar A.G.J.M. (2003). Liquid-based cervical cytology. Cancer.

[16] Ferenczy A., Robitaille J., Franco E., Arseneau J., Richart R.M., Wright T.C. (1996). Conventional cervical cytologic smears vs. ThinPrep smears. A paired comparison study on cervical cytology. Acta Cytol..

[17] Taylor S., Kuhn L., Dupree W., Denny L., De Souza M., Wright T.C. (2006). Direct comparison of liquid-based and conventional cytology in a South African screening trial. Int. J. Cancer.

[18] Arbyn M., Bergeron C., Klinkhamer P., Martin-Hirsch P., Siebers A.G., Bulten J. (2008). Liquid compared with conventional cervical cytology: A systematic review and meta-analysis. Obstet. Gynecol..

[19] Jeronimo J., Bansil P., Lim J., Peck R., Paul P., Amador J.J., Mirembe F., Byamugisha J., Poli U.R., Satyanarayana L. (2014). A multicountry evaluation of careHPV testing, visual inspection with acetic acid, and papanicolaou testing for the detection of cervical cancer. Int. J. Gynecol. Cancer.

[20] Gilham C., Sargent A., Kitchener H.C., Peto J. (2019). HPV testing compared with routine cytology in cervical screening: Long-term follow-up of ARTISTIC RCT. Health Technol. Assess..

[21] Maver P.J., Poljak M. (2020). Primary HPV-based cervical cancer screening in Europe: Implementation status, challenges, and future plans. Clin. Microbiol. Infect..

[22] Dillner J., Rebolj M., Birembaut P., Petry K.-U., Szarewski A., Munk C., de Sanjose S., Naucler P., Lloveras B., Kjaer S. (2008). Long term predictive values of cytology and human papillomavirus testing in cervical cancer screening: Joint European cohort study. BMJ.

[23] Pileggi C., Flotta D., Bianco A., Nobile C.G.A., Pavia M. (2014). Is HPV DNA testing specificity comparable to that of cytological testing in primary cervical cancer screening? Results of a meta-analysis of randomized controlled trials. Int. J. Cancer.

[24] Diamantis A., Magiorkinis E., Koutselini H. (2009). Fine-needle aspiration (FNA) biopsy: Historical aspects. Folia Histochem. Cytobiol..

[25] Swanton C. (2012). Intratumor heterogeneity: Evolution through space and time. Cancer Res..

[26] Crowley E., Di Nicolantonio F., Loupakis F., Bardelli A. (2013). Liquid biopsy: Monitoring cancer-genetics in the blood. Nat. Rev. Clin. Oncol..

[27] Palmirotta R., Lovero D., Cafforio P., Felici C., Mannavola F., Pellè E., Quaresmini D., Tucci M., Silvestris F. (2018). Liquid biopsy of cancer: A multimodal diagnostic tool in clinical oncology. Ther. Adv. Med. Oncol..

[28] Poulet G., Massias J., Taly V. (2019). Liquid Biopsy: General Concepts. Acta Cytol..

[29] Vasseur A., Kiavue N., Bidard F., Pierga J., Cabel L. (2021). Clinical utility of circulating tumor cells: An update. Mol. Oncol..

[30] Du K., Huang Q., Bu J., Zhou J., Huang Z., Li J. (2020). Circulating Tumor Cells Counting Act as a Potential Prognostic Factor in Cervical Cancer. Technol. Cancer Res. Treat..

[31] Tewari K.S., Sill M.W., Monk B.J., Penson R.T., Moore D.H., Lankes H.A., Ramondetta L.M., Landrum L.M., Randall L.M., Oaknin A. (2020). Circulating Tumor Cells In Advanced Cervical Cancer: NRG Oncology—Gynecologic Oncology Group Study 240 (NCT 00803062). Mol. Cancer Ther..

[32] Han K., Zou J., Zhao Z., Baskurt Z., Zheng Y., Barnes E., Croke J., Ferguson S.E., Fyles A., Gien L. (2024). Clinical Validation of Human Papilloma Virus Circulating Tumor DNA for Early Detection of Residual Disease After Chemoradiation in Cervical Cancer. J. Clin. Oncol..

[33] Cheng F., Su L., Qian C. (2016). Circulating tumor DNA: A promising biomarker in the liquid biopsy of cancer. Oncotarget.

[34] Zhang Y., Yao Y., Xu Y., Li L., Gong Y., Zhang K., Zhang M., Guan Y., Chang L., Xia X. (2021). Pan-cancer circulating tumor DNA detection in over 10,000 Chinese patients. Nat. Commun..

[35] (2017). Integrated genomic and molecular characterization of cervical cancer. Nature.

[36] Gu Y., Wan C., Qiu J., Cui Y., Jiang T., Zhuang Z. (2020). Circulating HPV cDNA in the blood as a reliable biomarker for cervical cancer: A meta-analysis. PLoS ONE.

[37] Sivars L., Hellman K., Crona Guterstam Y., Holzhauser S., Nordenskjöld M., Falconer H., Palsdottir K., Tham E. (2022). Circulating cell-free tumor human papillomavirus DNA is a promising biomarker in cervical cancer. Gynecol. Oncol..

[38] Song P., Wu L.R., Yan Y.H., Zhang J.X., Chu T., Kwong L.N., Patel A.A., Zhang D.Y. (2022). Limitations and opportunities of technologies for the analysis of cell-free DNA in cancer diagnostics. Nat. Biomed. Eng..

[39] Li L., Tong Y., Wu J., Xu X. (2023). Clinical applications and utility of ctDNA in cervical cancer and its precursor lesions: From screening to predictive biomarker. Cancer Cell Int..

[40] Li J., Lan X. (2023). Perspective on new cell-free DNA technologies for early cancer detection. Cancer Biol. Med..

[41] Bryan S.J., Lee J., Gunu R., Jones A., Olaitan A., Rosenthal A.N., Cutts R.J., Garcia-Murillas I., Turner N., Lalondrelle S. (2023). Circulating HPV DNA as a Biomarker for Pre-Invasive and Early Invasive Cervical Cancer: A Feasibility Study. Cancers.

[42] Cheung K.W.E., Choi S.R., Lee L.T.C., Lee N.L.E., Tsang H.F., Cheng Y.T., Cho W.C.S., Wong E.Y.L., Wong S.C.C. (2019). The potential of circulating cell free RNA as a biomarker in cancer. Expert Rev. Mol. Diagn..

[43] Pao C.C., Hor J.J., Yang F.P., Lin C.Y., Tseng C.J. (1997). Detection of human papillomavirus mRNA and cervical cancer cells in peripheral blood of cervical cancer patients with metastasis. J. Clin. Oncol..

[44] Tseng C.-J., Pao C.C., Lin J.-D., Soong Y.-K., Hong J.-H., Hsueh S. (1999). Detection of Human Papillomavirus Types 16 and 18 mRNA in Peripheral Blood of Advanced Cervical Cancer Patients and Its Association With Prognosis. J. Clin. Oncol..

[45] Reddi K.K., Holland J.F. (1976). Elevated serum ribonuclease in patients with pancreatic cancer. Proc. Natl. Acad. Sci. USA.

[46] Rapisuwon S., Vietsch E.E., Wellstein A. (2016). Circulating biomarkers to monitor cancer progression and treatment. Comput. Struct. Biotechnol. J..

[47] Berti F.C.B., Salviano-Silva A., Beckert H.C., de Oliveira K.B., Cipolla G.A., Malheiros D. (2019). From squamous intraepithelial lesions to cervical cancer: Circulating microRNAs as potential biomarkers in cervical carcinogenesis. Biochim. Biophys. Acta-Rev. Cancer.

[48] Peng Y., Croce C.M. (2016). The role of MicroRNAs in human cancer. Signal Transduct. Target. Ther..

[49] Acunzo M., Romano G., Wernicke D., Croce C.M. (2015). MicroRNA and cancer—A brief overview. Adv. Biol. Regul..

[50] Lin S., Gregory R.I. (2015). MicroRNA biogenesis pathways in cancer. Nat. Rev. Cancer.

[51] Hayashita Y., Osada H., Tatematsu Y., Yamada H., Yanagisawa K., Tomida S., Yatabe Y., Kawahara K., Sekido Y., Takahashi T. (2005). A polycistronic microRNA cluster, miR-17-92, is overexpressed in human lung cancers and enhances cell proliferation. Cancer Res..

[52] Di Leva G., Croce C.M. (2013). miRNA profiling of cancer. Curr. Opin. Genet. Dev..

[53] Friedman R.C., Farh K.K.-H., Burge C.B., Bartel D.P. (2009). Most mammalian mRNAs are conserved targets of microRNAs. Genome Res..

[54] Sharifi M., Salehi R., Gheisari Y., Kazemi M. (2014). Inhibition of microRNA miR-92a induces apoptosis and necrosis in human acute promyelocytic leukemia. Adv. Biomed. Res..

[55] Calin G.A., Dumitru C.D., Shimizu M., Bichi R., Zupo S., Noch E., Aldler H., Rattan S., Keating M., Rai K. (2002). Frequent deletions and down-regulation of micro- RNA genes miR15 and miR16 at 13q14 in chronic lymphocytic leukemia. Proc. Natl. Acad. Sci. USA.

[56] Shai A., Brake T., Somoza C., Lambert P.F. (2007). The human papillomavirus E6 oncogene dysregulates the cell cycle and contributes to cervical carcinogenesis through two independent activities. Cancer Res..

[57] Tornesello M.L., Faraonio R., Buonaguro L., Annunziata C., Starita N., Cerasuolo A., Pezzuto F., Tornesello A.L., Buonaguro F.M. (2020). The Role of microRNAs, Long Non-coding RNAs, and Circular RNAs in Cervical Cancer. Front. Oncol..

[58] He Y., Lin J., Ding Y., Liu G., Luo Y., Huang M., Xu C., Kim T., Etheridge A., Lin M. (2016). A systematic study on dysregulated microRNAs in cervical cancer development. Int. J. Cancer.

[59] Pardini B., De Maria D., Francavilla A., Di Gaetano C., Ronco G., Naccarati A. (2018). MicroRNAs as markers of progression in cervical cancer: A systematic review. BMC Cancer.

[60] Ning R., Meng S., Wang L., Jia Y., Tang F., Sun H., Zhang Z., Zhang C., Fan X., Xiao B. (2021). 6 Circulating miRNAs can be used as Non-invasive Biomarkers for the Detection of Cervical Lesions. J. Cancer.

[61] Kotani K., Iwata A., Kukimoto I., Nishio E., Mitani T., Tsukamoto T., Ichikawa R., Nomura H., Fujii T. (2022). Nomogram for predicted probability of cervical cancer and its precursor lesions using miRNA in cervical mucus, HPV genotype and age. Sci. Rep..

[62] Mori M.A., Ludwig R.G., Garcia-Martin R., Brandão B.B., Kahn C.R. (2019). Extracellular miRNAs: From Biomarkers to Mediators of Physiology and Disease. Cell Metab..

[63] Lawrie C.H., Gal S., Dunlop H.M., Pushkaran B., Liggins A.P., Pulford K., Banham A.H., Pezzella F., Boultwood J., Wainscoat J.S. (2008). Detection of elevated levels of tumour-associated microRNAs in serum of patients with diffuse large B-cell lymphoma. Br. J. Haematol..

[64] Sato-Kuwabara Y., Melo S.A., Soares F.A., Calin G.A. (2015). The fusion of two worlds: Non-coding RNAs and extracellular vesicles—Diagnostic and therapeutic implications (Review). Int. J. Oncol..

[65] Chen X., Ba Y., Ma L., Cai X., Yin Y., Wang K., Guo J., Zhang Y., Chen J., Guo X. (2008). Characterization of microRNAs in serum: A novel class of biomarkers for diagnosis of cancer and other diseases. Cell Res..

[66] Turchinovich A., Burwinkel B. (2012). Distinct AGO1 and AGO2 associated miRNA profiles in human cells and blood plasma. RNA Biol..

[67] Xu L., Yang B.-F., Ai J. (2013). MicroRNA transport: A new way in cell communication. J. Cell. Physiol..

[68] Chen X., Liang H., Zhang J., Zen K., Zhang C.-Y. (2012). Secreted microRNAs: A new form of intercellular communication. Trends Cell Biol..

[69] Wozniak A.L., Adams A., King K.E., Dunn W., Christenson L.K., Hung W.-T., Weinman S.A. (2020). The RNA binding protein FMR1 controls selective exosomal miRNA cargo loading during inflammation. J. Cell Biol..

[70] Groot M., Lee H. (2020). Sorting Mechanisms for MicroRNAs into Extracellular Vesicles and Their Associated Diseases. Cells.

[71] Villarroya-Beltri C., Gutiérrez-Vázquez C., Sánchez-Cabo F., Pérez-Hernández D., Vázquez J., Martin-Cofreces N., Martinez-Herrera D.J., Pascual-Montano A., Mittelbrunn M., Sánchez-Madrid F. (2013). Sumoylated hnRNPA2B1 controls the sorting of miRNAs into exosomes through binding to specific motifs. Nat. Commun..

[72] Santangelo L., Giurato G., Cicchini C., Montaldo C., Mancone C., Tarallo R., Battistelli C., Alonzi T., Weisz A., Tripodi M. (2016). The RNA-Binding Protein SYNCRIP Is a Component of the Hepatocyte Exosomal Machinery Controlling MicroRNA Sorting. Cell Rep..

[73] Chung J.-H., Eng C. (2005). Nuclear-Cytoplasmic Partitioning of Phosphatase and Tensin Homologue Deleted on Chromosome 10 (PTEN) Differentially Regulates the Cell Cycle and Apoptosis. Cancer Res..

[74] Teng Y., Ren Y., Hu X., Mu J., Samykutty A., Zhuang X., Deng Z., Kumar A., Zhang L., Merchant M.L. (2017). MVP-mediated exosomal sorting of miR-193a promotes colon cancer progression. Nat. Commun..

[75] Kosaka N., Iguchi H., Yoshioka Y., Takeshita F., Matsuki Y., Ochiya T. (2010). Secretory mechanisms and intercellular transfer of microRNAs in living cells. J. Biol. Chem..

[76] Kosaka N., Iguchi H., Hagiwara K., Yoshioka Y., Takeshita F., Ochiya T. (2013). Neutral Sphingomyelinase 2 (nSMase2)-dependent Exosomal Transfer of Angiogenic MicroRNAs Regulate Cancer Cell Metastasis. J. Biol. Chem..

[77] Koppers-Lalic D., Hackenberg M., Bijnsdorp I.V., van Eijndhoven M.A.J., Sadek P., Sie D., Zini N., Middeldorp J.M., Ylstra B., de Menezes R.X. (2014). Nontemplated nucleotide additions distinguish the small RNA composition in cells from exosomes. Cell Rep..

[78] Simpson R.J., Lim J.W., Moritz R.L., Mathivanan S. (2009). Exosomes: Proteomic insights and diagnostic potential. Expert Rev. Proteom..

[79] Kalluri R., LeBleu V.S. (2020). The biology, function, and biomedical applications of exosomes. Science.

[80] Chung I.-M., Rajakumar G., Venkidasamy B., Subramanian U., Thiruvengadam M. (2020). Exosomes: Current use and future applications. Clin. Chim. Acta.

[81] Zhang L., Yu D. (2019). Exosomes in cancer development, metastasis, and immunity. Biochim. Biophys. Acta-Rev. Cancer.

[82] Jeppesen D.K., Zhang Q., Franklin J.L., Coffey R.J. (2023). Extracellular vesicles and nanoparticles: Emerging complexities. Trends Cell Biol..

[83] Nabet B.Y., Qiu Y., Shabason J.E., Wu T.J., Yoon T., Kim B.C., Benci J.L., DeMichele A.M., Tchou J., Marcotrigiano J. (2017). Exosome RNA Unshielding Couples Stromal Activation to Pattern Recognition Receptor Signaling in Cancer. Cell.

[84] Yu W., Hurley J., Roberts D., Chakrabortty S.K., Enderle D., Noerholm M., Breakefield X.O., Skog J.K. (2021). Exosome-based liquid biopsies in cancer: Opportunities and challenges. Ann. Oncol. Off. J. Eur. Soc. Med. Oncol..

[85] Li P., Kaslan M., Lee S.H., Yao J., Gao Z. (2017). Progress in Exosome Isolation Techniques. Theranostics.

[86] He C., Zheng S., Luo Y., Wang B. (2018). Exosome theranostics: Biology and translational medicine. Theranostics.

[87] Wang W., Luo J., Wang S. (2018). Recent Progress in Isolation and Detection of Extracellular Vesicles for Cancer Diagnostics. Adv. Healthc. Mater..

[88] An M., Wu J., Zhu J., Lubman D.M. (2018). Comparison of an Optimized Ultracentrifugation Method versus Size-Exclusion Chromatography for Isolation of Exosomes from Human Serum. J. Proteome Res..

[89] Patel G.K., Khan M.A., Zubair H., Srivastava S.K., Khushman M., Singh S., Singh A.P. (2019). Comparative analysis of exosome isolation methods using culture supernatant for optimum yield, purity and downstream applications. Sci. Rep..

[90] Honegger A., Schilling D., Bastian S., Sponagel J., Kuryshev V., Sültmann H., Scheffner M., Hoppe-Seyler K., Hoppe-Seyler F. (2015). Dependence of Intracellular and Exosomal microRNAs on Viral E6/E7 Oncogene Expression in HPV-positive Tumor Cells. PLoS Pathog..

[91] Ma G., Song G., Zou X., Shan X., Liu Q., Xia T., Zhou X., Zhu W. (2019). Circulating plasma microRNA signature for the diagnosis of cervical cancer. Cancer Biomark..

[92] Zheng M., Hou L., Ma Y., Zhou L., Wang F., Cheng B., Wang W., Lu B., Liu P., Lu W. (2019). Exosomal let-7d-3p and miR-30d-5p as diagnostic biomarkers for non-invasive screening of cervical cancer and its precursors. Mol. Cancer.

[93] Lv A., Tu Z., Huang Y., Lu W., Xie B. (2020). Circulating exosomal miR-125a-5p as a novel biomarker for cervical cancer. Oncol. Lett..

[94] Churchman S.A., Moss J.A., Baum M.M. (2016). Accurate measurement of female genital tract fluid dilution in cervicovaginal lavage samples. J. Chromatogr. B.

[95] Okutucu B., Dınçer A., Habib Ö., Zıhnıoglu F. (2007). Comparison of five methods for determination of total plasma protein concentration. J. Biochem. Biophys. Methods.

[96] Dezzutti C.S., Hendrix C.W., Marrazzo J.M., Pan Z., Wang L., Louissaint N., Kalyoussef S., Torres N.M., Hladik F., Parikh U. (2011). Performance of Swabs, Lavage, and Diluents to Quantify Biomarkers of Female Genital Tract Soluble Mucosal Mediators. PLoS ONE.

[97] Liu J., Sun H., Wang X., Yu Q., Li S., Yu X., Gong W. (2014). Increased Exosomal MicroRNA-21 and MicroRNA-146a Levels in the Cervicovaginal Lavage Specimens of Patients with Cervical Cancer. Int. J. Mol. Sci..

[98] Zhang J., Liu S., Luo X., Tao G., Guan M., Yuan H., Hu D. (2016). Exosomal Long Noncoding RNAs are Differentially Expressed in the Cervicovaginal Lavage Samples of Cervical Cancer Patients. J. Clin. Lab. Anal..

[99] Chernyshev V.S., Chuprov-Netochin R.N., Tsydenzhapova E., Svirshchevskaya E.V., Poltavtseva R.A., Merdalimova A., Yashchenok A., Keshelava A., Sorokin K., Keshelava V. (2022). Asymmetric depth-filtration: A versatile and scalable method for high-yield isolation of extracellular vesicles with low contamination. J. Extracell. Vesicles.

[100] Nagamitsu Y., Nishi H., Sasaki T., Takaesu Y., Terauchi F., Isaka K. (2016). Profiling analysis of circulating microRNA expression in cervical cancer. Mol. Clin. Oncol..

[101] Liu P., Xin F., Ma C.F. (2015). Clinical significance of serum miR-196a in cervical intraepithelial neoplasia and cervical cancer. Genet. Mol. Res..

[102] Zhang Y., Zhang D., Wang F., Xu D., Guo Y., Cui W. (2015). Serum miRNAs panel (miR-16-2*, miR-195, miR-2861, miR-497) as novel non-invasive biomarkers for detection of cervical cancer. Sci. Rep..

[103] Sun L., Jiang R., Li J., Wang B., Ma C., Lv Y., Mu N. (2017). MicoRNA-425-5p is a potential prognostic biomarker for cervical cancer. Ann. Clin. Biochem. Int. J. Lab. Med..

[104] Wang J., Liu Y., Wang X., Li J., Wei J., Wang Y., Song W., Zhang Z. (2018). MiR-1266 promotes cell proliferation, migration and invasion in cervical cancer by targeting DAB2IP. Biochim. Biophys. Acta-Mol. Basis Dis..

[105] Farzanehpour M., Mozhgani S.-H., Jalilvand S., Faghihloo E., Akhavan S., Salimi V., Azad T.M. (2019). Serum and tissue miRNAs: Potential biomarkers for the diagnosis of cervical cancer. Virol. J..

[106] Qiu H., Liang D., Liu L., Xiang Q., Yi Z., Ji Y. (2020). A Novel Circulating MiRNA-Based Signature for the Diagnosis and Prognosis Prediction of Early-Stage Cervical Cancer. Technol. Cancer Res. Treat..

[107] Wang H., Zhang D., Chen Q., Hong Y. (2019). Plasma expression of miRNA-21, -214, -34a, and -200a in patients with persistent HPV infection and cervical lesions. BMC Cancer.

[108] Yang D., Zhang Q. (2019). miR-152 may function as an early diagnostic and prognostic biomarker in patients with cervical intraepithelial neoplasia and patients with cervical cancer. Oncol. Lett..

[109] Ocadiz-Delgado R., Lizcano-Meneses S., Trejo-Vazquez J., Conde-Perezprina J., Garrido-Palmas F., Alvarez-Rios E., García-Villa E., Ruiz G., Illades-Aguiar B., Leyva-Vázquez M.A. (2021). Circulating miR-15b, miR-34a and miR-218 as promising novel early low-invasive biomarkers of cervical carcinogenesis. APMIS.

[110] Wei H., Wen-Ming C., Jun-Bo J. (2017). Plasma miR-145 as a novel biomarker for the diagnosis and radiosensitivity prediction of human cervical cancer. J. Int. Med. Res..

[111] Zheng S., Li R., Liang J., Wen Z., Huang X., Du X., Dong S., Zhu K., Chen X., Liu D. (2020). Serum miR-638 Combined with Squamous Cell Carcinoma-Related Antigen as Potential Screening Biomarkers for Cervical Squamous Cell Carcinoma. Genet. Test. Mol. Biomark..

[112] Yamanaka Z., Sasaki T., Yamanaka A., Kato K., Nishi H. (2021). Circulating and tissue miR-100 acts as a potential diagnostic biomarker for cervical cancer. Cancer Biomark..

[113] Xin F., Liu P., Ma C.-F. (2016). A circulating serum miRNA panel as early detection biomarkers of cervical intraepithelial neoplasia. Eur. Rev. Med. Pharmacol. Sci..

[114] Wang W.-T., Zhao Y.-N., Yan J.-X., Weng M.-Y., Wang Y., Chen Y.-Q., Hong S.-J. (2014). Differentially expressed microRNAs in the serum of cervical squamous cell carcinoma patients before and after surgery. J. Hematol. Oncol..

[115] Jia W., Wu Y., Zhang Q., Gao G., Zhang C., Xiang Y. (2015). Expression profile of circulating microRNAs as a promising fingerprint for cervical cancer diagnosis and monitoring. Mol. Clin. Oncol..

[116] Jiang W., Pan J.-J., Deng Y.-H., Liang M.-R., Yao L.-H. (2017). Down-regulated serum microRNA-101 is associated with aggressive progression and poor prognosis of cervical cancer. J. Gynecol. Oncol..

[117] You W., Wang Y., Zheng J. (2015). Plasma miR-127 and miR-218 Might Serve as Potential Biomarkers for Cervical Cancer. Reprod. Sci..

[118] Ma Q., Wan G., Wang S., Yang W., Zhang J., Yao X. (2014). Serum microRNA-205 as a novel biomarker for cervical cancer patients. Cancer Cell Int..

[119] Tang B.-B., Liu S.-Y., Zhan Y., Wei L.-Q., Mao X.-L., Wang J., Li L., Lu Z.-X. (2015). microRNA-218 expression and its association with the clinicopathological characteristics of patients with cervical cancer. Exp. Ther. Med..

[120] Li C., Zheng X., Li W., Bai F., Lyu J., Meng Q.H. (2018). Serum miR-486-5p as a diagnostic marker in cervical cancer: With investigation of potential mechanisms. BMC Cancer.

[121] Kawai S., Fujii T., Kukimoto I., Yamada H., Yamamoto N., Kuroda M., Otani S., Ichikawa R., Nishio E., Torii Y. (2018). Identification of miRNAs in cervical mucus as a novel diagnostic marker for cervical neoplasia. Sci. Rep..

[122] Kong Q., Tang Z., Xiang F., Jiang J., Yue H., Wu R., Kang X. (2017). Diagnostic Value of Serum hsa-mir-92a in Patients with Cervical Cancer. Clin. Lab..

[123] Yu J., Wang Y., Dong R., Huang X., Ding S., Qiu H. (2012). Circulating microRNA-218 was reduced in cervical cancer and correlated with tumor invasion. J. Cancer Res. Clin. Oncol..

[124] Rasi Bonab F., Baghbanzadeh A., Ghaseminia M., Bolandi N., Mokhtarzadeh A., Amini M., Dadashzadeh K., Hajiasgharzadeh K., Baradaran B., Bannazadeh Baghi H. (2021). Molecular pathways in the development of HPV-induced cervical cancer. EXCLI J..

[125] Ferlay J., Shin H., Bray F., Forman D., Mathers C., Parkin D.M. (2010). Estimates of worldwide burden of cancer in 2008: GLOBOCAN 2008. Int. J. Cancer.

[126] Shah S., Senapati S., Klacsmann F., Miller D., Johnson J., Chang H.-C., Stack M. (2016). Current Technologies and Recent Developments for Screening of HPV-Associated Cervical and Oropharyngeal Cancers. Cancers.

